# Service user reflections on the impact of involvement in research

**DOI:** 10.1186/s40900-018-0095-1

**Published:** 2018-03-26

**Authors:** Jim Gordon, Sue Franklin, Sabrina A. Eltringham

**Affiliations:** 1 Directorate of Therapeutics and Palliative Care, Sheffield Teaching Hospitals NHS Foundation Trust, Glossop Road, Sheffield, S10 2JF UK; 2 Faculty of Health and Wellbeing, Collegiate Crescent, Sheffield Hallam University, Sheffield, S10 2BP UK

**Keywords:** Service user, Impact, Involvement

## Abstract

**Plain English summary:**

Public involvement can impact on research, on the public who give advice, on the researchers and the research participants. Evaluating impact is an important part of the research process. Two members of a hospital-based patient research panel and our coordinator have written this paper. Our panel covers a range of rehabilitation and palliative services. These services form the “Therapeutics and Palliative Care Directorate”. We describe how we worked collaboratively with hospital staff and co-produced questionnaires to evaluate the impact of our involvement. We compared the different perspectives of the researchers and panel members on our contribution to the research. We present evidence from these different standpoints, including how our panel made a difference. We found we needed to adapt how we collected the views of the researchers and our members to ensure it was meaningful to our group whilst delivering the wider objective of the hospital. A key finding has been how our involvement has extended into other groups, which has identified opportunities for sharing resources and experience, including areas such as cost effectiveness. Our two-person membership of a high level Board of Academics and Senior Clinicians, which oversees the research we contribute to, has resulted in our opinions influencing the heart of the Directorate’s research strategy. We have learned the importance of a flexible approach as the Directorate changes, and the demands on us grow. This will continue to help us share our own development, successes and experience and extend the benefits from working this way.

**Abstract:**

**Background**

Reports about the impact of patient and public involvement in research can be improved by involving patients and research staff more collaboratively to co-produce instruments to measure their involvement. This commentary, written by two members of a hospital-based patient panel and their coordinator for its work, describes how we co-produced instruments to evaluate the impact and effectiveness of our involvement. We present here the results, including our quantitative and qualitative findings, of this patient led evaluation and reflect on how our involvement has made a difference to the research projects and research infrastructure within the hospital in which we operate and on us as a panel.

**Methods**

Existing impact frameworks and guidelines were reviewed. Members co-produced and piloted qualitative questionnaires to identify values associated with patient and public involvement (PPI) from both a researcher and panel member perspective, and collected quantitative metrics to provide descriptive statistics on the type of involvement and activities. Members also produced a comments slip to provide contemporaneous feedback after each meeting.

**Results**

The panel has reviewed 36 research projects for the Therapeutics and Palliative Care Directorate drawn from speech and language therapy, physiotherapy, occupational therapy, dietetics, podiatry, palliative care services and chaplaincy. Some of the main results of our involvement have been the development of grant applications and making written information more understandable for research participants. Examples of how the Panel made a difference included providing an effective forum for debate by providing practical suggestions to improve research design and identifying potential issues that may not have occurred to the researcher. The panel has had an impact outside of meetings both within the context in which it operates and on the individuals involved. Examples included: influencing the Directorate research agenda, sharing resources with other groups, developing research relationships, and enabling member participation in different roles and settings.

**Discussion**

Embedding ourselves within the Directorate research infrastructure has enabled us to adapt to organisational change and actively contribute to the research strategy. There is greater scope for involvement in areas of cost effectiveness and economic evaluation. Increasing member contributions and networking with other groups provides added value as well as cross fertilisation of ideas as part of our widening impact.

**Conclusion**

Evaluating the impact of our involvement has improved our understanding of what aspects of involvement work best for the panel and the researchers who attend our meetings, and in the different settings that we work in. It has helped us to focus on how we need to develop to maximise our resources going forward.

## Introduction

Patient and Public Involvement (PPI) can have a range of impacts. It can impact on research, on the members of the public who contributed, on the researchers and research participants, and on the wider community [1]. Increasing evidence about the impact of PPI from the perspective of service users is emerging [2]. However accounts of impact can be improved by providing more information about the context and mechanism of involvement [3] and involving service users collaboratively to develop instruments to measure impact [4]. This commentary describes how members of a patient research panel in a large National Health Service (NHS) Teaching Hospital worked collaboratively with the Trust Research Department and other patient research panels in the hospital to co-produce tools to evaluate the impact of patient involvement from the different perspectives of the researcher and the panel member. We present the results from the evaluation of our panel and discuss how our involvement to date has made a difference to the research projects and research infrastructure within the hospital in which we operate and on us as a panel.

The Therapeutics and Palliative Care Patient Research Panel launched in May 2014 as part of the Directorate’s investment in its research infrastructure and commitment to integrating service users into the research process. The Directorate provides a wide range of diagnostic, rehabilitation and palliative care services, and supports patients with a broad spectrum of acute, chronic and progressive health conditions and diseases. Members were selected based on their direct experience of these services or indirect experience as carers of patients who use these services. These services include speech and language therapy, physiotherapy, occupational therapy, dietetics, psychology, chaplaincy, tissue viability and palliative care services. Many of our members are active users of these services or care for someone who uses these services so their experiences are contemporaneous. The panel meets quarterly, but also offers the facility for members to contribute online for those who can not attend meetings to provide feedback and for researchers the opportunity to have access to the panel between meetings.

The main aim of the panel through face-to-face meetings and providing online feedback between meetings is to ensure that the research being carried out is high quality and patient focused. Members are involved throughout the research process from helping to prioritise research topics; offering feedback on research proposals including applications for ethical approval; providing ideas to improve patient recruitment; to disseminating the findings to the wider public. Another important part of our remit is to raise staff awareness of PPI, our panel, and the value of involving patients and the public in research. We are actively involved in educational and training activities and events. Additionally two members of our panel sit on a high level board of academics and senior clinicians, which oversees the research we contribute to, where they have the opportunity to influence the Directorate’s research strategy.

Evaluating impact is an important part of the research process [5]. Having contributed to the Directorate’s research for two and half years the panel wanted to know if they had made a difference and felt they had sufficient amount of data to form the basis of an evaluation. As well as being good practice to measure our intervention it was felt an evaluation might improve our involvement by learning from the feedback of researchers as well as our members. The decision to evaluate our activities also coincided with a Trust wide initiative to measure the impact of all its panel activities.

## Methods

Our co-ordinator attended a meeting with the chairs and co-ordinators of the other hospital patient research panels and the Trust’s Research Department to discuss how the impact of the panels should be measured and evaluated. Existing impact frameworks and guidelines [1, 6] were reviewed and we co produced with the other hospital panels and the Research Department separate qualitative questionnaires for panel members and researchers. The aim of the questionnaires was to identify the values associated with PPI from a patient and researcher perspective. It was felt important that as part of the impact framework we should also capture data for example on the frequency and level of our input on research projects. Information in the form of quantitative data was collected by our co-ordinator to provide descriptive statistics on panel membership, type of input into the research process, attendance at local and regional events, and staff educational activities.

Our members piloted the new panel member questionnaire and the researcher feedback questionnaire. We commented individually and collectively and our feedback was taken back to the joint chair and co-ordinator working party. Amendments to both questionnaires were collectively agreed.

Researchers who attend the Therapeutic and Palliative Care panel meetings are asked to complete their questionnaire after each meeting. The panel questionnaire is designed for annual feedback. In addition to the Trust questionnaire our panel also felt it important to introduce a comments slip to be completed at the end of each meeting. This was in response to some members finding it difficult to recall information over twelve months and wanted to be able to implement change more quickly. The comments slip asks panel members what aspects of the meeting did they find most and least rewarding, and how they feel they made a difference.

## Results

### Quantitative metrics

**Fig. 1:**
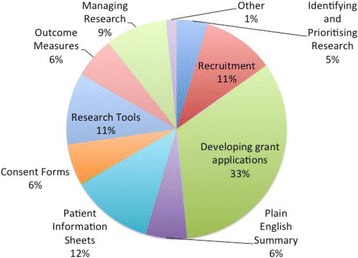
Type of involvement (May 2014 to March 2017)

The Panel has 14 members and has reviewed 36 research proposals between May 2014 and March 2017. A total of 33 researchers attended meetings or approached the panel remotely and a number requested feedback more than once. The main areas which the panel provides input is in the design of studies: recruitment of participants, developing grant applications, reviewing participant materials (for example, information sheets and consent forms), commenting on data collection tools (for example, questionnaires and interview topic guides) and outcome measures (Fig. 1).

Other types of involvement include prioritising topics for future research and providing input at a strategic level, for example by reviewing and commenting on the Directorate research strategy. Changes to the research strategy included refocusing the strategy to more strongly reflect the primary aim of undertaking research for patient benefit, and including patient relevant outcome measures. The Directorate achieved Trust Academic Status in 2016. The review committee acknowledged clear evidence of the panel’s involvement. Other examples of involvement have included being a member of a project’s steering group, attending workshops, and disseminating their work at local, regional and national research and public engagement events. We have also presented at staff education and training events including a “Valuing patients in research” workshop, in which members of the panel presented the patient perspective. This educational event has since been adopted by the Trust Research Department and rolled out as part of their educational programme.

### Qualitative data

Researchers were asked what their perceptions were of presenting to the panel before and after the meeting. Most researchers before attending were aware that seeking the opinions of members of the public was an integral and valuable part of the research process and were optimistic about potential insights that may not have occurred to them. One researcher felt “apprehensive about being asked questions I couldn’t answer!” After the meeting researchers stated they felt the comments were constructive and supportive: “It encouraged me to persevere with my application”.

Overall 83% of researchers stated they found the panels comments very useful and 77% made changes as a consequence of the feedback. Eighty four per cent stated they were very satisfied with their experience and 94% said they would definitely use the panel again. Examples of how researchers found the panel’s comments very useful included consideration of the burden to research participants in a study about developing speech recognition software for patients with paralysis on breathing support machines. The panel suggested shortening the length of the voice recordings as part of the software development given potential for participant fatigue, which was favourably received by the research ethics committee. Another example of added value was for a podiatry research study about peripheral arterial disease funded by the National Centre for Sport and Exercise Medicine. The panel suggested changes on how to make the recruitment poster more impactful and altering the way the participant information sheet was written. It was felt some of the language in the information sheet could be less threatening and might put people off participating in the study. The researchers took on board the panel’s advice, changed the participant information sheet and the study achieved its recruitment target. An example given from the minority of researchers who did not find it useful included not having anyone with direct experience of the condition of the topic of their research for a study about a triage system using photographic technology for wound management. Although no panel members had any personal experience one of our members who is carer for her husband who has experience, was able to offer a carer point of view and was subsequently invited to join the project’s steering group.

The most important question for us as panel members and one we ensured was included in both the panel and researcher questionnaires, was how the panel had made a difference. Table 1 summarises the themes and practical examples from the panel and researchers on how the panel has made a difference to the research.

**Table 1:** How has the Panel made a difference?

Panel Perspective	Researcher Perspective
Enhancing research proposals by providing practical suggestions to improve research design e.g. recruitment, sampling, treatment approaches, and outcome measures.	Highlighting the benefits and issues to be addressed e.g. selection bias, data collection tools, information governance issues, and wider involvement of service users.
Identifying potential issues that may not have occurred to the researcher.	Roles and training of members of the research team e.g. conducting interviews.
Giving a patient perspective and experience e.g. customising materials to improve recruitment, participant burden, and planned intervention.	Adapting methods of data collection e.g. consistency of language, number and length of interviews, and focusing on key outcome measures.
Improving information accessibility for research participants e.g. lay summaries, participant information sheets, consent forms, and recruitment materials.	Being inclusive e.g. using more easily understandable language, avoiding acronyms, creating aphasia friendly information to avoid exclusion.
Reinforcing the importance of the research.	Prompted us to seek funding following service user feedback.
Providing an effective forum for debate; concentrating on key elements from the patient’s perspective.	Reinforced patient engagement and using a co-design methodology.

As a panel we felt we enhanced research proposals by identifying issues from a user’s perspective that may not have occurred to researchers. Members felt they improved research studies by proposing suggestions to minimise selection bias of participants, encouraging a wider approach to intervention and outcome measures, and recommending customisation of the recruitment materials to help recruit and retain participants, and the use of more patient friendly language in information sheets. On a personal level some members of our panel felt their involvement had strengthened their knowledge of the research process and enabled them to continue to be part of a professional or academic community, which they had to prematurely withdraw from due to their condition or carer commitments. Other personal benefits included feeling they were giving something back to the National Health Service.

Suggestions on how the panel could be improved included: increasing the diversity of its membership from different social and cultural groups and to target recruitment with direct and indirect experience of different conditions and services to continue to reflect the breadth of patients which the Directorate provides services for. Researchers liked the range of ways they could contact the panel through the co-ordinator and requested if this could extend beyond the panel meeting. A request was made for longer discussion time for certain research items.

## Discussion

### Adapting to changes in the context of the research environment

What we choose to say as panel members, and how well we fulfil our role, relates to our ability to know and learn about the organisation and the research context. This has been challenging as the “organisation” has changed around us. The composition of the Directorate’s services has evolved and we are now part of a larger Care Group in the Trust. This has had implications for our workload and the experience and abilities of panel members to remain useful and effective. Adaptation has been successfully achieved through the panel and the Directorate working together through regular consultation on the changing infrastructure and its likely significance for the skills and workload of the panel. Trust staff have adopted a shared information approach and thus fully engaging us in the process as well as research proposal content. For example, the Research Lead for the Directorate attends panel meetings and a regular item of the agenda is scheduled where members can discuss the implications of organisational change with the opportunity to discuss our ability and willingness to respond. The result for us has been heightened motivation and enthusiasm to adapt including our involvement in panel recruitment and adjusting the member mix. Thus all have worked to maintain our panel’s strong cross condition contribution.

### Areas for further involvement

In our view much of the findings and metrics in this paper are due to our wide remit. We have been strongly encouraged to question and advise and this has had a significant impact on the measurement and improvement of outcomes likely to be more acceptable, meaningful and understandable for patients. However we also now see greater scope for our panel involvement in strengthening research at the input or research design stage particularly in respect to methodology, especially where this would contribute in the important areas of cost effectiveness and economic evaluation.

These may be more challenging and difficult areas for researchers to bring to the Panel but if consideration of cost and cost-effectiveness are not more frequently part of what researchers consider appropriate for panels - and we find seldom do at present - then we ourselves are not being used to maximum effectiveness. The panel can play a role in ensuring cost-effective approaches or treatments are not missed and are made available to patients. One of the co-authors of this paper has been influenced in this view through simultaneous membership of a PPI group for a study about adaptive design clinical trials and the impact on the economic evaluation of healthcare technologies [7]. This is also an example where members extend their involvement and create links with other patient panels and cross-fertilise ideas. In going forward we are keen to ensure that such future opportunities are not missed.

### Setting the research agenda

The panel has been able to influence the research agenda through representation on the board of academics and senior clinicians and contribution to the Directorate’s Research Strategy 2015–2018. This helped it gain Academic Status. At an inter disciplinary level using a workshop format, we have provided a patient perspective in working with speech and language therapists in narrowing down competing priorities to a manageable number for their future research program. We envisage such strategic contributions developing and expanding. We have been invited to consider producing a panel led view on research and establishing panel generated ideas for a research agenda.

### Impact on individuals and the group

The experience has been a very rewarding one in the main. There has been a sense of building working relationships and partnerships between Trust staff and ourselves that have been primarily positive and open. We have concentrated on giving ideas and sharing information as opposed to focusing on what is wrong or appearing to criticise. Without holding back on any concerns, we feel we have gained confidence in contributing constructively. Whilst striving to maintain objectivity, we have appreciated the insight we have been allowed into the challenges of the NHS environment. Finally there is gaining considerable satisfaction in being able to “give something back” through helping staff improve their proposals and secure approval of funding. These reflections are consistent with some of the practices of appreciative enquiry [8] in particular where stakeholders such as us are engaged to determine change by focusing on what is working well and doing more of it.

### Future development and suggestions for improvement

We suggest there is much added value from investment in the panel through maintaining or increasing the contribution some members are able to make outside the panel. This is important for the transference of ideas and knowledge these contributions can create as well as forging mutually beneficial relationships. This includes dissemination through participation in public engagement events and more interaction with researchers and involvement in the research process itself including membership of steering groups for research trials or being a subject or volunteer in a study.

This evaluation has identified the importance attached to maintaining the appropriate gender, age and cultural balance within the panel as well as the diversity of the conditions, which the Directorate serves. We have recognised this as a future priority for us to assist with.

An illustration of the importance the Directorate attaches to our involvement is our representation on the academic board, including the Chair. Although now well established, we see this relationship very much developing in the future: we play a full part in all discussions at Board level and in turn report to the board as PPI progresses and is evaluated. Indeed it was as a result of this that at Board professorial level it was suggested that we write this paper and put it forward for publication as a PPI outcome and discussion document worth sharing with others.

Finally as we gain further experience we anticipate greater value added from patient panels working together to gain maximum benefit from our time commitment and the NHS resource investment in our support. The involvement of our members in other groups has opened up a network of local, regional and national links, which is a vital and reciprocal resource for improving patient focussed research and outcomes, and one which we will continue to foster.

## Conclusion

Our involvement in the development of tools to evaluate our impact has helped ensure that measurements are meaningful from a service user perspective, and outcomes important to this group are included. In addition the results from the evaluation has improved our understanding of what aspects of our involvement work best across different contexts in which we operate, whether providing feedback to a researcher about the accessibility of their recruitment materials on a particular researcher project; to influencing the Directorate’s research agenda. It has also highlighted areas for improvement. This will help sharpen our focus on how we need to develop membership and activities to maximise future resource investment in our remit and activities. In addition we feel service user involvement in the provision of advice about cost and other methodological implications important to NHS decision making should be prioritised and that sharing our learning through greater networking with other groups is needed to maximise our own value, effectiveness and the resource justification for PPI.

## Abbreviations

NHS: National Health Service
PPI: Patient and Public Involvement
